# ﻿*Pothosdeleonii* (Araceae, Potheae, Pothoideae), a new species from Bukidnon, Mindanao, Philippines

**DOI:** 10.3897/phytokeys.247.130721

**Published:** 2024-10-15

**Authors:** Maria Melanie P. Medecilo-Guiang, Derek Cabactulan

**Affiliations:** 1 Center for Biodiversity Research and Extension in Mindanao, Central Mindanao University, University Town, Musuan, Maramag, Bukidnon 8714, Philippines; 2 Plant Biology Division, Institute of Biological Sciences, College of Arts and Sciences, Central Mindanao University, University Town, Musuan, Maramag, Bukidnon 8714, Philippines; 3 48 Corrales and 1st Streets, Nazareth, Cagayan de Oro City, Misamis Oriental 9000, Philippines

**Keywords:** Allopothos, Araceae, aroid diversity, critically endangered, endemic, *Pothosphilippinensis*

## Abstract

A new species of *Pothos* is described and illustrated. This species is very similar to *Pothosphilippinensis* (sheathing and leaf morphology) but differs by the inflorescence and flowers. It is closely related also to *P.kingii* by the deep purple inflorescence, but differs in having longer peduncle, broadly ovate-concave to cucullate spathe (which is deep wine-red when fresh to purplish-black when senescing), and the spadix that is 7/10^th^ the entire length of the spathe.

## ﻿Introduction

*Pothos* L. (Araceae Juss., Pothoideae, Potheae ; Mayo et al. 1997; Cusimano et al. 2011; Chartier et al. 2014; Haigh et al. 2022) is a genus comprising 65 species (WCPV 2024) distributed in subtropical to tropical regions from Madagascar to Oceania, China to Australia with Borneo as the center of diversity (Boyce 2000; Boyce and Hay 2001; Wong and Boyce 2019). Morphologically, it includes forest-dwelling hemiepiphytes (Zots 2013; Zots 2020; Zots et al. 2021a, 2021b) with bisexual flowers (Linnaeus 1753). Recently, a number of new species were recorded in Asia (Nguyen et al. 2017; Wong and Boyce 2019; Rajkumar et al. 2020; Wong et al. 2020) and the latest is the new combination, *P.venustus* (Wall. ex C.DC.) A.Hay & P.C.Boyce in Malesia (Hay and Boyce 2022).

In the Philippines, a total of eight species [*P.cylindricus* C. Presl, *P.dolichophyllus* Merr., *P.inaequilaterus* (C. Presl) Engl., *P.insignis* Engl., *P.luzonensis* (C. Presl) Schott, *P.ovatifolius* Engl., *P.philippinensis* Engl., and *P.scandens* L.] have been recorded (Boyce and Hay 2001) of which four are endemic (Pelser et al. 2011). Two species (*P.insignis* and *P.ovatifolius*) are confined in Borneo, Sulawesi, Sumatera, Malay Peninsula, and Philippines, while *P.scandens* is widely distributed in Asia and other South-East Asian regions. The last complete revision of *Pothos* including the Philippine species was published by Boyce and Hay (2001). Subsequently, no additional species of *Pothos* from the Philippines has been published.

During bird expeditions in 2019 by Dr. Miguel De Leon, he observed a species of aroid with very striking inflorescence in one of the Robert S. Kennedy Bird Conservancy conservation sites in Bukidnon. The plant was collected because of its unique and regal inflorescence. Last February of 2024, this new species was again observed and photographed *in situ*. The original collection and fresh material were sent for further examination by the authors. Morphologically, the observed plants are distinct and do not match with any described species of *Pothos*. As a consequence, we here propose to describe a new species for science.

## ﻿Material and methods

The voucher specimens were collected from the type locality last February 18, 2024 with Gratuitous Permit R-102024-22. Specimens were processed using the methods of Bridson and Forman (1999) and deposited at the herbaria **PNH**, and **CMUH** (acronyms according to Thiers 2024, continuously updated). Detailed photographs and a description were taken from fresh materials in the field using a digital camera. Taxonomic identification was based on morphological vegetative and reproductive characters following the aforementioned literature.

## ﻿Taxonomic treatment

### ﻿ Pothos deleonii

Taxon classificationPlantaeAlismatalesAraceae

M.P.Medecilo-Guiang & D.Cabactulan sp. nov.

42CC663E-7D1E-5E8B-8C5B-1A85090A6E4E

urn:lsid:ipni.org:names:77350292-1

Figs 1 , 2

#### Type.

Philippines • Bukidnon Province, Manolo Fortich date: February 18, 2024, MPMG1005 (holotype PNH! PNH259023); isotypes CMUH!)

#### Diagnosis.

This new species is morphologically similar to *Pothosphilippinensis* based on vegetative characters but differs by the purplish peduncle, dark wine red to purplish black spathe and pendulous peduncle and acuminate to caudate (vs. acuminate to apiculate) leaf apex. *P.deleonii* is closest to *R.kingii* and *P.atropurpurascens* M. Hotta by having a purple cylindrical spadix but differs from 2 later species by having a much longer, purplish green to dark purple peduncle, 16–18 cm long (vs 5 cm long in *P.kingii* and 8 cm in *P.atropurpurascens*) broadly ovate spathe, subsessile spadix and 7/10 the entire length of the spathe, bigger diameter of flowers (2 mm) and flower orientation.

**Figure 1.**
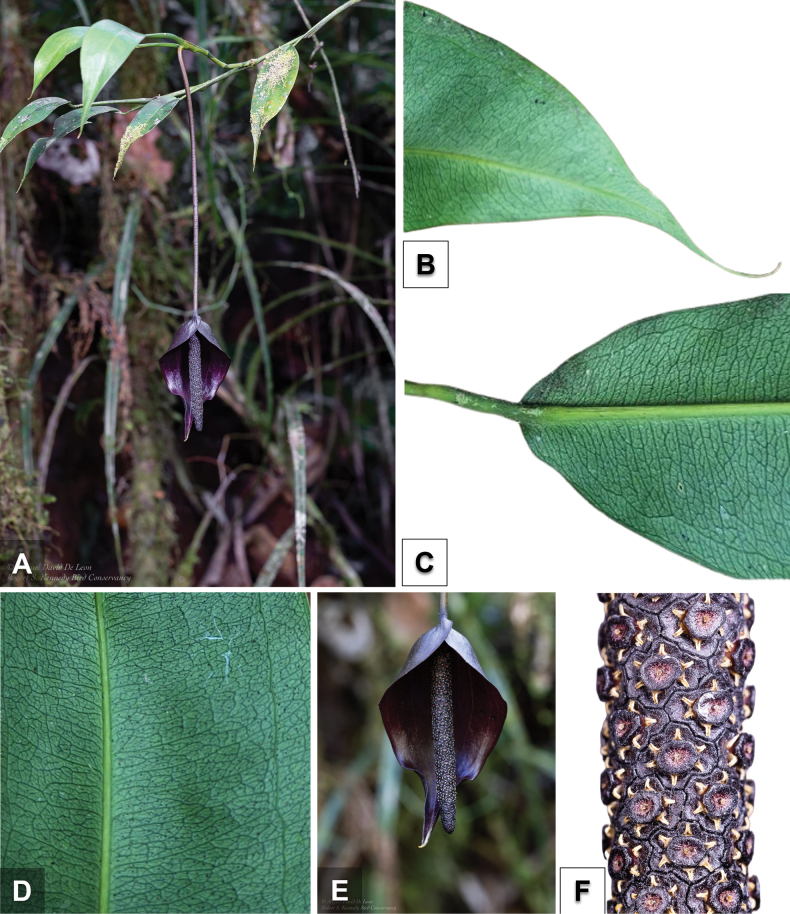
*Pothosdeleonii* M.P Medecilo-Guiang and D. Cabactulan **A** habit with flowering branch **B** leaf apex **C** leaf base **D** venation pattern **E** inflorescence **F** detail of spadix.

#### Description.

Plant growth glabrous, root climbing, fibrous liana. **Stem** moderate slender, slightly woody, terete, green, internodes 2 cm long by 7 mm in diameter in juvenile stage and 7 cm long by 3 mm in diameter upon maturity, younger shoots arising above from the base of the half of the entire length of older stem. **Roots** present along stem when juvenile, fewer to absent towards the terminal shoots with inflorescence. **Leaves** spreading, long petiolate, petiole slender angular, 45 degree angled towards the stem, 6–15 cm long by 4–12 mm in diameter, petioles deeply canaliculate with petiolar sheath prominently distinct, erect extending to pulvinus and imbricate to the stem, base decurrent, apex prominently geniculate, petiolar sheath apically ligulate in young growth, ligule and sheath margins later scarious-disintegrating, 7–14 cm long by 0.6–2.5 mm wide, 7/8 of the entire length of the petiole. **Lamina** oblong-lanceolate, occasionally falcate, asymmetrical, coriaceous, margins entire, flattened, shallowly sinuate to slightly conduplicate, 15.5–26.3 cm long by 4–6 cm wide, apex acuminate to caudate and thickened, base acute to obtuse, slightly cordate in juvenile stage, adaxially slightly glossy, dark green and abaxially pale green. Venation closely pinnate, brochidodromous, midrib pale green, adaxially flattened and abaxially rounded raised, primary lateral veins arising running at the base of midrib towards the intra-marginal veins, secondary veins arising at each primary lateral vein, close-spaced disorganized reticulated towards the intra-marginal vein, 2 intra-marginal veins on both sides of the leaf, arising from the base of midrib towards the apex, disorganized reticulated, primary intra-margins 6.5–9.0 mm wide, secondary intra-margins 0.8–1.0 mm wide, venation adaxially less prominent to absent when fresh and slightly present when dry, abaxially prominent visible in both fresh and dry state, intra-marginal veins raised abaxially when dry, outer intramarginal vein remaining very close and parallel to margin. **Inflorescence** solitary, elongated, deflexed, pendent, and arising from each terminal of matured stem, positive geotropic. **Peduncle** long terete, stout, deflexed, base purplish-green, dark purple towards the base of the spathe, 16–18 cm long by 2–3 mm in diameter. **Spathe** broadly ovate, concave to cucullate, apex acuminate, curved, base cordate and slightly decurrent on the peduncle, dark wine red when fresh and purplish black when near wilting, 9.2–10.0 cm long and 4.5–5.5 cm wide, softly-leathery when fresh and papery when dried, prominently 9-nerved, veins dirty white when fresh and dark purplish black when dried, less prominent acrodomous venation. **Spadix** long cylindrical, stout, subsessile, dark purplish, 6.4–6.9 cm long by 3.5–4.0 mm in diameter, 7/10 the entire length of the spathe, flower c. 2.7 mm, tepals 1 mm by 0.70 mm, oblong-cymbiform, dark purplish-black, apex fornicate, triangular, truncate, minutely 3–4-lobed, flower compressed angular-ellipsoid, black purplish, stylar region truncate, centrally depressed, 1.5 mm in diameter, stigma prominently punctiform, stamens, 0.3 mm long by 0.1 mm wide, filaments strap-shaped, thecae c. 0.2 mm in diameter, creamy yellow, ovary 1.0–1.6 mm high by 0.25–0.70 mm in diameter, fertile zone 5.2–5.8 cm long, appendix 8–11 mm long. Infructescence not observed.

**Figure 2.**
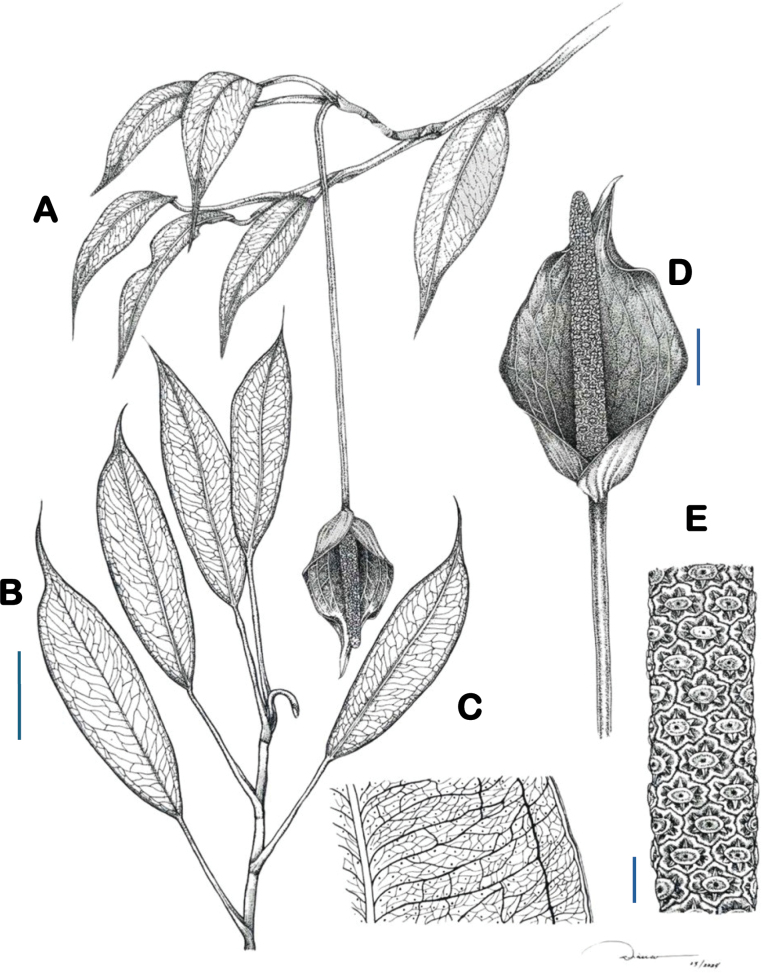
Line drawing of *Pothosdeleonii***A** whole plant **B** leaf phyllotaxy **C** venation pattern **D** Inflorescence **E** detail of spadix: Scale bars: 10 mm (**B**); 15 mm (**D**); 5 mm (**E**).

#### Ecology and habitat.

Known only in a highly restricted area in two sites in degraded secondary, open-canopy dipterocarp forest in a 500-hectare avian conservation site located at the northern foothills of Mt. Kitanglad, 1,150 and 1,270 m a s l. The plant grows from the ground or at the base of trees (*Shorea* sp.) and trunk of *Alsophila* sp., 12–20 cm dbh; adherent to host plants from the ground to 4–6 meters, at which plants become branched and grow freely, either pendent or supported by vines or branches of adjacent trees. No specific association with plants other than host plants can be gleaned. But the habitat is favorable to other aroids like *Alocasiasanderiana*, *A.zebrina*, and *Rhaphidophora* sp.

#### Distribution.

Philippines. BUKIDNON: Manolo Fortich, (exact location withheld as a conservation measure).

#### Vernacular.

None recorded.

#### Etymology.

The specific epithet honors Dr. Miguel David De Leon, a vitreoretinal surgeon and wildlife conservationist, who first photographed the species *in situ*, and supports the conservation of wild flora, particularly orchids and hoyas, and fauna, specifically raptors, in Mindanao.

#### Uses.

No known usage in traditional medicine; potentially valuable in horticulture.

#### Conservation status.

Extensive survey in the 500-hectare area revealed only 12 mature individuals. Because the plants are located in an avian conservation site and constantly monitored by the Robert S. Kennedy Bird Conservancy, there are currently no threats. However, due to its small population and highly restricted extent of occurrence (less than 2 hectares), this species is considered as Critically Endangered based on the criteria of IUCN (2024).

#### Notes.

*P.deleonii* belongs to subgenus Allopothos under the *Pothosbarberianus* group and it is closely related to the Philippine endemic *P.philippinensis* and two Malaysian species: *P.kingii* and *P.atropurpurascens* but differs in the size, shape, color and orientation of other floral segments (Table 1).

**Table 1.** Morphological comparison of *P.deleonii*, *P.philippinensis*, *P.kingii* and *P.atropurpurascens*.

	*Pothosdeleonii*	*P.philippinensis*	*P.kingii*	*P.atropurpurascens*
**Leaves**				
**Color**	dark green	drying dull greenish brown	bright to mid-green	midgreen
**midrib**	pale green	slightly paler green	slightly paler green	pale yellow
**blade shape**	adaxially flattened and adaxially raised	ovate to oblong elliptic or narrowly lanceolate	prominently raised	slightly raised
**base**	oblong lanceolate, occasionally falcate	rounded, rarely truncate or cordate	ovate to elliptic or lanceolate	ovate-elliptic to narrowly oblong elliptic or oblanceolate
**apex**	acute to obtuse, slightly cordate	acute to long attenuate	acute to rounded	acute to obtuse
**primary lateral**	acuminate to caudate	apiculate	acute or attenuate	rounded and abruptly cuspidate, minutely apiculate
**veins**	arising at 85–90°	arising at c. 45°	45–65°	65–80°
**Peduncle**	deflexed ca. 90° at base	nodding to deflexed	reflexing held inverted	reflexed c. 90° at base
**Spathe shape**	broadly ovate concave to cucullate	triangular, subcucullate	ovate, deeply cucullate	ovate, deeply cucullate
**apex**	acuminate	long-acuminate	acute	acute to attenuate
**color**	dark wine red to dark purple	pale green	deep purple inside and out	white stained purple
**Spadix**	subsessile	sessile but basally decurrent on spathe	sessile	sessile
**color**	dark purple	paler green	deep purple-brown	deep purple gray
**Flowers**	ca. 2.7 mm	c. 1.3 mm	1 mm	1.2 mm

## Supplementary Material

XML Treatment for Pothos deleonii

## ﻿Additional information

### Author ORCIDs

Maria Melanie P. Medecilo-Guiang https://orcid.org/0000-0003-1584-1585

Derek Cabactulan https://orcid.org/0009-0001-4087-5595

### Data availability

All of the data that support the findings of this study are available in the main text.
